# Exploratory factor analysis of the Dizziness Handicap Inventory (German version)

**DOI:** 10.1186/1472-6815-10-3

**Published:** 2010-03-15

**Authors:** Annette Kurre, Caroline HG Bastiaenen, Christel JAW van Gool, Thomas Gloor-Juzi, Eling D de Bruin, Dominik Straumann

**Affiliations:** 1 Department of Rheumatology and Institute of Physical Medicine, University Hospital Zurich, Switzerland; 2 Maastricht University, school CAPHRI, Department of Epidemiology and Faculty of Health Medicine and Life Sciences, the Netherlands; 3 Institute of Human Movement Sciences and Sport, ETH, Zurich, Switzerland; 4 Interdisciplinary Center for Vertigo & Balance Disorders, Departments of ENT, Neurology & Psychiatry, University Hospital Zurich, Switzerland

## Abstract

**Background:**

The Dizziness Handicap Inventory (DHI) is a validated, self-report questionnaire which is widely used as an outcome measure. Previous studies supported the multidimensionality of the DHI, but not the original subscale structure. The objectives of this survey were to explore the dimensions of the Dizziness Handicap Inventory - German version, and to investigate the associations of the retained factors with items assessing functional disability and the Hospital Anxiety and Depression Scale (HADS). Secondly we aimed to explore the retained factors according to the International Classification of Functioning, Disability and Health (ICF).

**Methods:**

Patients were recruited from a tertiary centre for vertigo, dizziness or balance disorders. They filled in two questionnaires: (1) The DHI assesses precipitating physical factors associated with dizziness/unsteadiness and functional/emotional consequences of symptoms. (2) The HADS assesses non-somatic symptoms of anxiety and depression. In addition, patients answered the third question of the University of California Los Angeles-Dizziness Questionnaire which covers the impact of dizziness and unsteadiness on everyday activities. Principal component analysis (PCA) was performed to explore the dimensions of the DHI. Associations were estimated by Spearman correlation coefficients.

**Results:**

One hundred ninety-four patients with dizziness or unsteadiness associated with a vestibular disorder, mean age (standard deviation) of 50.6 (13.6) years, participated. Based on eigenvalues greater one respectively the scree plot we analysed diverse factor solutions. The 3-factor solution seems to be reliable, clinically relevant and can partly be explained with the ICF. It explains 49.2% of the variance. Factor 1 comprises the effect of dizziness and unsteadiness on emotion and participation, factor 2 informs about specific activities or effort provoking dizziness and unsteadiness, and factor 3 focuses on self-perceived walking ability in relation to contextual factors. The first factor correlates moderately with disability and the HADS (values ≥0.6). The second factor is comparable with the original physical subscale of the DHI and factors retained in previous studies.

**Conclusions:**

The results of the present survey can not support the original subscale structure of the DHI. Therefore only the total scale should be used. We discuss a possible restructuring of the DHI.

## Background

The Dizziness Handicap Inventory (DHI) is a validated, self-report questionnaire designed to evaluate the precipitating physical factors associated with dizziness and unsteadiness as well as the functional and emotional consequences of vestibular disease [1]. This questionnaire has gained wide acceptance as a useful measure of disability resulting from dizziness and unsteadiness [2] and has been used as an outcome measure to evaluate the effects of non-medical, medical and surgical interventions for treating dizziness caused by many different diagnoses associated with the vestibular system [3].

Although Jacobson & Newman [1] developed the DHI already in 1990, the questionnaire fits well in the International Classification of Functioning, Disability and Health (ICF) endorsed by the WHO (2001) to provide a unifying framework for classifying the consequences of disease [4]. The ICF is structured around the four components: (1) body functions and structures, (2) activities and participation, (3) environmental factors, and (4) personal factors. The Classification is based on a bio-psycho-social model. Functioning and disability are viewed as complex interactions between the health condition of the individual and the contextual factors of the environment as well as personal factors [4]. The DHI not only collects the consequences of vestibular disorders on a wide spectrum of health domains, as classified by the ICF [5], but also gives an impression about the interaction between the four components in a single patient. For example, the analysis of the responses of a patient may show that the self-perceived ability of this person to walk depends on the environmental condition. Furthermore Jacobson & Newman [1] constructed their questionnaire in order to get an idea about cause and effect of the patient's perceived symptoms. While items of the physical subscale are thought to assess which activities (ICF component activities and participation) trigger dizziness or imbalance (ICF component body function), items of the functional and emotional subscale may assess the consequences of the symptoms on the degree of participation in social life and emotion, respectively (ICF components activities and participation and body function).

The internal validity of the content domains suggested by Jacobson and Newman was investigated for the original English [6], Spanish [7], and Dutch version [8]. The results of these studies support the multidimensionality of the DHI, but not the original subscale structure.

The primary objective of this study was to investigate the internal validity of the subscale structure and to explore the dimensions of the German version of the Dizziness Handicap Inventory (DHI-G). Since the DHI assesses disability and feelings of anxiety and depression, the associations of (i) the DHI, (ii) the original subscales and (iii) the factors retained by the exploratory factor analysis with single items assessing functional disability and the Hospital Anxiety and Depression Scale (HADS) [9] are investigated. We hypothesized that the measures/items assessing functional disability and those assessing emotion correlate moderately (0.51 - 0.75) [10]. Secondly we aimed to match DHI items with ICF categories to investigate whether the retained factors could be explained with the ICF.

## Methods

### Participants

Patients who had suffered for at least one month from vertigo, dizziness or unsteadiness were included in the study. Problems had to be associated with a vestibular disorder. Further inclusion criteria were the ability to walk, to independently manage about 50% of the daily tasks, and to understand and speak German. Exclusion criteria were dizziness or unsteadiness exclusively due to cardiopulmonary, musculoskeletal, neurological or psychic disorders [5].

### Procedure

In the period between July 2007 and May 2009, participants were recruited from the Interdisciplinary Center for Vertigo & Balance Disorders, Departments of ENT, Neurology & Psychiatry at the University Hospital Zurich. Patients were referred to the center primarily for diagnostic reasons. The diagnostic procedure consisted of a detailed clinical history, a complete neuro-otological bedside examination, laboratory tests, and MR imaging of the brain with special emphasis on brainstem, cerebellum and vestibulo-cochlear nerves. All patients who fulfilled the inclusion criteria and not the exclusion criteria were included in the study, if they gave written consent. The ethics committee of the Canton of Zurich approved the study, which was the continuation of a previous survey on the reliability and validity of the DHI-G [5].

### Measures

The DHI is a 25-item questionnaire that was designed to help patients rate their self-perception of disability from dizziness [1]. A *yes *response gives a score of 4 points, *sometimes *2 points, and *no *0 points. The total score ranges from zero (no disability) to 100 (severe disability). The scale consists of a 7-item physical subscale, a 9-item emotional subscale, and a 9-item functional subscale. Several validated translations and cross-cultural adaptations of the DHI exist. All language versions showed good internal consistency and test-retest reliability [2,5,11-14].

The 14-item Hospital Anxiety and Depression Scale (HADS) assesses independently non-somatic symptoms of anxiety (HADS-A) and depression (HADS-D). Each item is rated with 0 - 3 points. Scores on the two subscales range from zero (no sign of anxiety or depression) to 21 (maximum level of anxiety or depression). 8 - 10 points indicate possible and > 10 points probable anxiety or depression [9]. The HADS is often used to assess patients with dizziness and unsteadiness, but is not validated in these patients. Kammerlind et al. (2005) investigated the test-retest reliability of the HADS in a sample of Swedish patients with vestibular disorders and reported acceptable Intraclass Correlation Coefficients (ICCs) [15].

In addition, patients rated their functional disability in one global question as mild, moderate or severe. They also answered question no. 3 of the 5-item University of California Los Angeles Dizziness Questionnaire (UCLA-DQ) [16]. This question concerns the impact of dizziness and unsteadiness on the patient's daily activities. This is rated by a Likert scale that ranges from 1 (least severe limitation) to 5 (most severe limitation). The reliability of the original UCLA-DQ is unknown. Kammerlind et al. (2005) reported acceptable ICCs for the Swedish version of the UCLA-DQ [15].

### Data Analysis

Baseline characteristics of the study population, such as mean age, gender distribution, the degree of disability, and neuro-otologic diagnoses were assessed. The distributions of DHI-G and HADS scores were statistically investigated.

To evaluate the different dimensions of the DHI a principal component analysis (PCA) was conducted. Before PCA was done, various assumptions on inter-correlations of the 25 DHI items were tested [17]: The Determinant has to be > 0.00001 and Bartlett's test highly significant (p < 0.001). The Kaiser-Meyer-Olkin (KMO) measure of sampling adequacy for all, as well as the individual variables, was set at > 0.75.

We opted to extract factors with eigenvalues greater than 1 [Kaiser's criterion (K1)] and to repeat the PCA after inspection of the scree plot, a graph plotting each eigenvalue against the factor [17]. The cut-off point for selecting factors should be at the point of inflexion of this curve [18]. We chose a factor-solution after analysing the interpretability and estimating the reliability of the retained factors. Per factor, four variables are the minimum [19] and at least four factor loadings have to be greater than 0.6 [20]. With communalities in the 0.5 range, samples between 100 and 200 can be good enough [21].

We conducted the PCA with oblique rotation and interpreted primarily the pattern matrix [17]. To assess the fit of the factor models, we looked at the differences between the observed correlations and the model-based correlations. No more than 50% of the residuals should be greater than 0.05 [17].

We estimated the associations of the factors and the original subscales with 1) the items assessing functional disability and 2) the HADS by calculating Spearman's correlation coefficients. Coefficients < 0.25 were considered to indicate weak associations, 0.26 - 0.50 fair, 0.51 - 0.75 moderate and ≥0.76 strong associations [10].

The internal consistency of the retained factors was investigated by estimating Cronbach's alpha coefficients and corrected item-total correlations (CI-TCs).

The analyses were computed using the SPSS version 16.0 computer software.

## Results

### Patient characteristics

One hundred ninety-four patients with a mean (standard deviation) age of 50.6 (13.6) years were included. Characteristics of the study population are summarized in Table 1.

**Table 1:** Baseline characteristics of the study population

Characteristics of the patients	*n *= 194
Age (mean [SD], range) (yr)	50.6 (13.6) 21 - 77
Sex (n, [%])	
Male	74 (38.1)
Female	120 (61.9)
Groups of diagnosis (n [%])	
UPVD	72 (37.1)
BPVD	16 (8.2)
CVD	71 (36.6)
Multisensory/multifactorial	35 (18.0)
Duration of dizziness or unsteadiness (n [%])	
> 1 mo and maximum 6 mo	55 (28.4)
> 6 mo and maximum 12 mo	25 (12.9)
> 12 mo	114 (58.8)
Level of disability (n [%])	
Little	52 (26.8)
Moderate	98 (50.5)
Severe	44 (22.7)
Limitation in activity respectively participation (UCLA; Question 3) (n [%])	
No effect at all	14 (7.2)
Continuing out all activities but with allowance for the dizziness	36 (18.6)
Continuing most of the activities	78 (40.2)
Continuing some of the activities	49 (25.3)
Unable to continue any of the activities	17 (8.8)
DHI-G total scale^a^	
Mean (SD)	44.8 (22.2)
Median (range)	44 (0 - 93)
Functional subscale^b^	
Mean (SD)	16.7 (9.7)
Median (range)	18 (0 - 36)
Physical subscale^c^	
Mean (SD)	13.9 (7.0)
Median (range)	14 (0 - 28)
Emotional subscale^b^	
Mean (SD)	14.1 (8.8)
Median (range)	14 (0 - 36)
HADS^d^	
Mean (SD)	11.5 (7.7)
Median (range)	10 (0 - 33)
Anxiety subscale^e^	
Mean (SD)	6.3 (4.3)
Median (range)	6 (0 - 17)
Depression subscale^e^	
Mean (SD)	5.1 (4.2)
Median (range)	4 (0 - 18)

BPVD indicates bilateral peripheral vestibular dysfunction; CVD, central vestibular dysfunction; DHI-G, Dizziness Handicap Inventory - German version; HADS, Hospital Anxiety Depression Scale; multisensory/multifactorial causes of dizziness; SD, standard deviation; UCLA-DQ, University of California Los Angeles - Dizziness Questionnaire; UPVD, unilateral peripheral vestibular dysfunction;

^a ^Maximum score of the DHI-G: 100 points; higher scores mean more disability

^b ^Maximum scores of the functional and emotional subscale: 36 points

^c ^Maximum score of the physical subscale: 28 points

^d ^Maximum score of the HADS: 42 points; higher scores mean more anxiety or depression

^e ^Maximum scores of the anxiety and depression subscale: 21 points

### Factor analysis

Exploring the correlation matrix proved the variables to be suited for a factor analysis. The Determinant resulted in a value of 7.62E-006 which is slightly under the recommended value. Bartlett's test was highly significant (*p *< 0.0001). The KMO resulted in a value of 0.89. Twenty-three Measures of Sampling Adequacy were greater than 0.8, three greater than 0.7.

**Figure 1:**
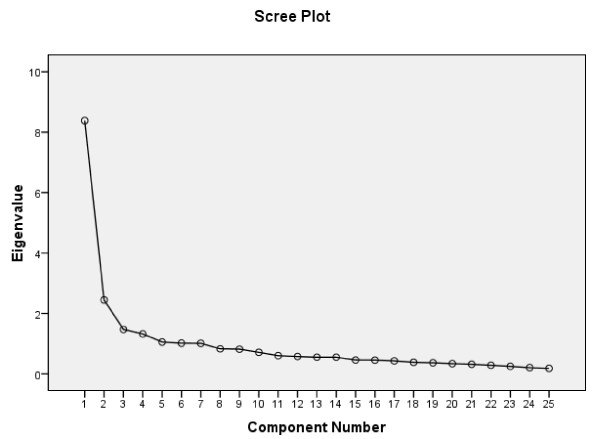
**The Screeplot is a graph plotting each eigenvalue against the factor**. The initial eigenvalues of factor 1, 2, 3 and 4 are 8.381, 2.447, 1.469 and 1.321. After oblique rotation the values are in the 4- factor solution 6.202, 4.040, 5.350 and 3.044 and in the 3- factor solution 6.035, 4.252 and 6.080.

The K1-criterion resulted in a 7-factor solution explaining 66.8% of the variance. Because 4 of the 7 factors consisted of less than 4 variables this solution was not further investigated. The inspection of the scree plot indicated 4- and 3-factor solutions (Figure 1).

#### The Four-factor solution

The 4-factor solution explained 54.5% of the variance. The investigation of the fit of the model resulted in 129 (43%) non-redundant residuals. Communalities after factor extraction showed values in the range of 0.5, with F7 (difficulties in reading) having the lowest value (0.247).

The first factor consists of 9 items, 5 of these with factor loadings greater than 0.6 (Table 2). Six items belong to the original emotional and 3 to the original functional subscale. This factor describes "the effect of dizziness and unsteadiness on emotion and participation". The first two items with the highest factor loadings assess the feeling of being depressed (E23) and frustrated (E2). The items E21 (feeling handicapped) and E10 (feeling embarrassed in front of others) also assess emotional functions, classified within the ICF as impairments of body functions [4] (Additional file 1: Table S1), but in addition document the feeling of an individual in relation to the society. Such feelings may lead to avoiding social activities and consequently to restricted participation. Within the ICF participation restrictions are defined as problems, an individual experiences in involvement in life situations/life areas [4]. Problems in fulfilling the usual responsibilities in job and house (F24), limited participation in social activities (F6), stress in the relationship to the family or friends (E22), and restrictions in travelling (F3), are examples of participation restrictions. Difficulties in concentrating (E18), is a limitation of activity (Definition: activity is the execution of a task or action by an individual [4]). But problems in concentration may seriously affect the execution of daily tasks and reduce the capacity of an individual in the society. Therefore, this item also suits in this factor. The composition of items of this factor demonstrates a close relationship between an individual's emotion and participation. This was supported by the moderate associations of this factor with the single items assessing functional disability (*r *= 0.70 and 0.60), the HADS (0.6), and especially the HADS-D (0.68) (Table 3).

**Table 2:** The four factor solution of the principal component analysis

		Factor 4.1		Factor 4.2		Factor 4.3		Factor 4.4	
Item	Abbreviated item description	Effect of dizziness and unsteadiness on emotion and participation		Specific activities/movements provoking dizziness or unsteadiness		Contextual factors or effort provoking dizziness and unsteadiness		Dependence of others/fear	
		**load**	**CI-TC**	**Load**	**CI-TC**	**load**	**CI-TC**	**load**	**CI-TC**
E 23	feeling depressed	**0.788**	0.68						
E 2	feeling frustrated	**0.765**	0.60						
F 24	job/house responsibilities	**0.685**	0.71						
E 21	feeling handicapped	**0.663**	0.76						
E 10	embarrassed in front of others	**0.623**	0.38						
F 6	restriction of social activities	0.578	0.74						
E 22	stressed relationships	0.559	0.65						
F 3	restriction of travel	0.477	0.65						
E 18	difficulties in concentrating	0.452	0.49						
Cronbachs alpha			0.88						
									
P 13	turning over in bed			**0.824**	0.56				
F 5	getting into or out of bed			**0.751**	0.56				
P 1	looking up			**0.672**	0.49				
P 11	quick head movements			**0.656**	0.52				
P 25	bending over			0.589	0.49				
F 7	difficulties in reading	0.193		0.265	0.27	0.124		0.169	
Cronbachs alpha					0.74				
									
P 17	walking down a sidewalk					**0.738**	0.59		
F 19	walking around in dark					**0.715**	0.44		
E 15	afraid of appearing intoxicated					**0.603**	0.51		
P 8	ambitious activities like sports					0.539	0.63		
P 4	walking down a supermarket aisle					0.502	0.48		
F 12	avoid heights					0.482	0.48		
F 14	strenuous housework					0.412	0.64		
Cronbachs alpha							0.80		
									
E 20	afraid to stay home alone							**0.759**	0.52
E 9	afraid of leaving home alone							**0.707**	0.70
F 16	walking by yourself							**0.512**	0.62
Cronbachs alpha									0.77

The table indicates factor loadings, corrected item-total correlations (CI-TC) and Cronbachs alpha correlation coefficients estimated in n = 194. Values of factor loadings are results of the pattern matrix. Bold face indicates loadings with absolute values of 0.6 or more. Loadings < 0.4 are not reported with exception of the values of F7, which has only low loadings. The 4-factor solution explained 54.5% of the variance.

Abbreviations: E indicates emotional subscale; F, functional subscale; P, physical subscale of the Dizziness Handicap Inventory - German version; load indicates factor loading.

**Table 3:** Associations between the DHI, the original subscales, the retained factors, disability items and the HADS

	Level of disability	Limitation of daily activity/participation (UCLA-DQ3)	HADS	HADS-A	HADS-D
DHI-G	0.71**	0.55**	0.59**	0.43**	0.66**
E	0.63**	0.57**	0.62**	0.46**	0.67**
F	0.71**	0.51**	0.54**	0.39**	0.62**
P	0.46**	0.34**	0.36**	0.26**	0.40**
Factor 3.1	0.70**	0.60**	0.60**	0.43**	0.68**
Factor 3.2	0.53**	0.40**	0.35**	0.26**	0.39**
Factor 3.3	0.53**	0.34**	0.52**	0.39**	0.56**
Factor 4.1	0.70**	0.60**	0.60**	0.43**	0.68**
Factor 4.2	0.44**	0.35**	0.27**	0.22**	0.28**
Factor 4.3	0.53**	0.33**	0.47**	0.33**	0.55**
Factor 4.4	0.52**	0.42**	0.47**	0.37**	0.48**

Values are Spearman correlation coefficients: ** correlation is significant at 0.01 level (1-tailed); * correlation is significant at the 0.05 level (1-tailed). Bold face indicates moderate associations.

Abbreviations: DHI-G indicates Dizziness Handicap Inventory-German version; E, F and P, emotional, functional, and physical subscales of the DHI-G; Factor 3.1 - 3.3 indicate the 3 components of the 3-factor solution; Factor 4.1 - 4.4 the 4 components of the 4-factor solution; HADS, Hospital Anxiety and Depression Subscale; HADS-A, anxiety subscale of the HADS; HADS-D, depression subscale of the HADS; UCLA-DQ3, item 3 of the University of California Los Angeles -Dizziness Questionnaire

The second factor is composed of 6 variables, 4 of these with factor loadings > 0.6 (Table 2). This factor primarily assesses if specific activities, with typical movements of the head and body in space, provoke vertigo, dizziness or unsteadiness. Item F7 (difficulties in reading) which may indicate problems in vestibular-visual interaction, does not really fit in this factor. Furthermore the factor loading of F7 is below the recommended value of 0.36 [22].

The third factor comprises of 7 items, 3 of these with factor loadings > 0.6. This factor assesses how contextual factors or effort relate to dizziness, unsteadiness and self-perceived walking ability. Depending on the ability to process and differentially use afferent input, individuals may feel dizzy or unsteady when walking down a sidewalk (P17), walking around in the dark (F19), walking down a supermarket aisle (P4), or being in height (F12). In our opinion item E15, 'afraid of appearing intoxicated', also targets the self-perceived walking ability. Many patients who can not walk straight ahead affirm this question. According to the linking rules as described by Cieza et al. (2005) item P8 (ambitious activities like sport) and F14 (strenuous housework) target participation (sport: ICF-category d0201; doing housework: ICF-category d640) as well as body function (muscle power function: ICF category b730 [23] (Additional file 1: Table S1). In our opinion the decisive terms, Cieza et al. call it the "meaningful concepts", in these 2 questions are "ambitious" and "strenuous"[23]. Patients may ask themselves whether they have enough voluntary movement control, muscle power, or muscle endurance to perform sportive or strenuous activities. Under this assumption, the third factor assesses how contextual factors and impairments of body functions affect the performance of activities.

The fourth factor consists of 3 items, 2 with factor loadings > 0.6 (Table 2). Together, this makes the reliability of this factor questionable [19,20]. All 3 items assess the dependence of the patient on others, 2 of them in relation to anxiety.

The correlation coefficients show marginally moderate associations between the third and fourth factor and the self estimated level of disability (r = 0.53 and 0.52) and between the third factor and HADS-D (0.55) (Table 3). Factor 2 correlates only fair with the items assessing functional disability and weak with the HADS. These results support the impression that different dimensions are assessed by the four retained factors. The limited number of items of the fourth factor may be the reason for the fair correlation with HADS-D.

#### The three factor solution

The 3-factor solution explained 49.2% of the variance. The investigation of the fit of the model resulted in 137 (45%) non-redundant residuals. The inspection of the communalities showed values in the range of 0.5, with F7 (difficulties in reading) and E20 (afraid to stay home alone) having the lowest values (0.24, 0.26). The first factor is composed of the same items as factor 1 of the 4-factor solution (Table 4). The second factor comprises P8 (ambitious activities like sport) and F14 (strenuous housework) in addition to the items of factor 2 in the 4-factor solution. We therefore named the second factor "specific activities/movements or effort provoking dizziness and unsteadiness". The third factor includes the three items of the fourth factor of the 4-factor solution. According to ICF personal assistance, as well as environmental factors, belong to the contextual factors that influence the ability of an individual to perform activities or to be an active participator in life situations, respectively. Four of the 8 items directly ask for aspects of self-perceived walking ability in relation to contextual factors. In our clinical experience, the items E15 (afraid of appearing intoxicated) and E9 (afraid of leaving home alone) are closely related to walking ability. Therefore, we consider this factor targeting the dimension "self-perceived walking ability/feeling of postural stability in relation to contextual factors". Linking the DHI items to ICF-categories demonstrates the relevance of the contextual factors in the third factor by the high number of e-(environmental) categories (Additional file 1: Table S1) [23].

**Table 4:** The three factor solution of the principal component analysis

			Factor 3.1:		Factor 3.2:		Factor 3.3:	
Item	Abbreviated item description	Item mean (SD)	Effect of dizziness and unsteadiness on emotion and participation		Specific activities/movements or effort provoking dizziness or unsteadiness		self-perceived walking ability and feeling of postural stability in relation to contextual factors	
			**load**	**CI-TC**	**load**	**CI-TC**	**load**	**CI-TC**
E 23	feeling depressed	1.8 (1.5)	**0.792**	0.68				
E 2	feeling frustrated	2.4 (1.4)	**0.757**	0.59				
E 21	feeling handicapped	2.3 (1.5)	**0.653**	0.76				
F 24	job/house responsibilities	2.0 (1.6)	**0.652**	0.71				
E 10	embarrassed in front of others	1.6 (1.9)	**0.622**	0.38				
F 6	restriction of social activities	2.1 (1.7)	0.567	0.74				
E 22	stressed relationships	1.3 (1.6)	0.563	0.65				
F 3	restriction of travel	2.2 (1.7)	0.463	0.65				
E 18	difficulties in concentrating	1.9 (1.5)	0.397	0.49				
Cronbachs alpha				0.88				
								
P 13	turning over in bed	1.7 (1.6)			**0.747**	0.52		
P 11	quick head movements	2.9 (1.5)			**0.726**	0.55		
P 1	looking up	2.3 (1.6)			**0.707**	0.48		
F 5	getting into or out of bed	1.6 (1.6)			**0.683**	0.56		
P 25	bending over	2.0 (1.7)			**0.618**	0.48		
P 8	ambitious activities like sports	2.3 (1.7)			0.439	0.56		
F 14	strenuous housework	2.1 (1.6)			0.410	0.60		
F 7	difficulties in reading	1.5 (1.6)	0.200		0.253	0.35	0.209	
Cronbachs alpha						0.80		
								
P 17	walking down a sidewalk	1.1 (1.5)					**0.713**	0.58
F 19	walking around in dark	1.7 (1.7)					**0.701**	0.42
F 16	walking by yourself	1.2 (1.5)					**0.623**	0.61
E 15	afraid of appearing intoxicated	1.4 (1.7)					0.580	0.51
E 9	afraid of leaving home alone	1.0 (1.3)					0.572	0.58
P 4	walking down a supermarket aisle	1.8 (1.7)					0.553	0.51
F 12	avoid heights	2.3 (1.8)					0.543	0.43
E 20	afraid to stay home alone	0.5 (1.1)					0.416	0.44
Cronbachs alpha								0.79

The table indicates item statistics, factor loadings, corrected item-total correlations (CI-TC) and Cronbachs alpha correlation coefficients estimated in n = 194. Values of factor loadings are results of the pattern matrix. Bold face indicates loadings with absolute values of 0.6 or more. Loadings < 0.4 are not reported with exception of the values of F7, which has only low loadings. The 3-factor solution explained 49.2% of the variance.

Abbreviations: E indicates emotional subscale; F, functional subscale; P, physical subscale of the Dizziness Handicap Inventory - German version; load indicates factor loading.

Like in the 4-factor solution, the first factor shows moderate associations with the items assessing functional disability (*r *= 0.70 and 0.60), the HADS (0.60) and HADS-D (0.68) (Table 3). Marginally moderate associations can also be seen between the second and third factors and self estimated level of disability (0.53), as well as between factor 3 and the HADS (0.52) and HADS-D (0.56).

The 3 - factor solution seems to be the most reliable solution and holds clinical relevant dimensions. Cronbach alpha coefficients of the retained factors and corrected item-total correlations (CI-TCs) within each factor fulfil the commonly accepted minimal standards of 0.7 for Cronbachs alpha and 0.2 for CI-TCs [24,25] (Table 2 and 4).

## Discussion

The exploratory factor analysis of the German version of the Dizziness Handicap Inventory (DHI-G) led to a clinically interesting 3-factor solution which seems to be reliable with at least 8 variables per factor and twice 5- and once 3-factor loadings greater than 0.6. Although the three factors differ from the original 3 subscales, the objectives of the DHI to quantify the functional and emotional consequences of dizziness or imbalance as well as to assess symptom provoking activities are supported. While factor 1 assesses the effect of dizziness and unsteadiness on emotion and participation, factor 2 informs about specific activities/movements or effort provoking dizziness or unsteadiness, and factor 3 about self-perceived walking ability and the feeling of postural stability in relation to contextual factors. The dimension as targeted by factor 1 is supported by moderate associations with the Hospital Anxiety and Depression Scale and items assessing functional disability. The found 3-factor solution is clinically interesting. While the first factor might indicate whether health care professionals, such as social workers, occupational therapists or psychologists, should become involved in the assessment and treatment of the individual, the scores of the second and third factors might indicate whether a patient will benefit from therapy, primarily emphasizing physical or behavioural training.

The factor analyses of the English [6], Spanish [7] and Dutch versions [8] of the DHI all led to more than one factor solution, whereby most authors preferred the 3- or 4-factor solutions. Especially the 3-factor solutions show parallels (Additional file 2: Table S2): Factor 1 includes between 9 and 14 items. Five items (E23 'feeling depressed', E2 'feeling frustrated', E21 'feeling handicapped', F24 'job/house responsibilities', and F6 'restriction of social activities') are part of all first factors. E10 (embarrassed in front of others), E22 (stressed relationships), F3 (restriction of travel), and E18 (difficulties in concentrating) are included in the first factor of three versions. As shown in Additional file 2: Table S2 the descriptions of the first factors are comparable and focus mainly on participation restrictions.

Factor 2 is the most similar among all language versions. The number of enclosed items ranges between 2 and 8. P13 (turning over in bed) and F5 (getting into or out of bed) are included in all second factors. P11 (quick head movements), P25 (bending over) and P8 (ambitious activities like sports) are part of the second factor in three; P1 (looking up) and F14 (strenuous housework) are included in two language versions. Authors describe the dimension assessed by this factor primarily as limitations in specific activities in relation to motion sensitivity.

The common objective targeted by the third factor is the aspect of visuo-vestibular dysfunction and context dependent behaviour. P4 (walking down the supermarket aisle) is the item included in the third factor of all language versions. P17 (walking down a sidewalk), F19 (walking around in the dark), E15 (afraid of appearing intoxicated) and F12 (avoid heights) belong to this factor in three of the language versions. F16 (walking by yourself), E9 (afraid of leaving home alone) and E20 (afraid to stay home alone), all assessing the dependence of personal assistance, belong to factor 3 of the English and German version, and to factor 1 of the Dutch and Spanish version.

The differences in the results of the various factor analysis studies may be attributed to several factors. One important aspect is the study population. The sample sizes vary from 95 [6] to 337 individuals [7] (Additional file 2: Table S2). Our sample size fulfils the recommendations of MacCallum et al. [21] and Stevens [23] regarding the values of communalities and factor loadings. The samples also differ in the aetiology of dizziness and unsteadiness. In the sample of Perez et al., 125 (37.1%) of the individuals had Menière's disease, whereas in the sample of Vereeck et al. 104 (48.6%) of the individuals had a vestibular schwannoma. While individuals with Menière's disease typically suffer of unpredictable attacks of symptoms, individuals with vestibular schwannoma might have more continuous symptoms. Despite this diversity in the samples the 3-factor solutions of Perez et al. and Vereeck et al. are quite similar with respect to the first 2 factors (Additional file 2: Table S2). As mentioned before, the first factor of Perez and Vereeck and colleagues contains E9, F16 and E20 ("dependence of others"). This may be because patients with Menière's disease or vestibular schwannoma have an organic, mostly chronic and progressive disorder. Patients with Menière's disease have a high comorbidity of anxiety and depression disorders, and as a result frequently develop avoidance behaviour which can lead to participation limitations [26,27].

The most critical point and therefore a limitation of our study can be attributed to the nature of PCA. For PCA, one assumes that variables are numeric and normally distributed. Items of the DHI, however, are ordinal. We therefore repeated the factor analysis with a Categorical Principal Component Analysis (CATPCA) restricted to 4 and 3 factors. In both cases, the analysis resulted in a quasi 2-factor solution, with nearly all items - except P13 (turning over in bed), P11 (quick head movements), P1 (looking up), F5 (getting into or out of bed) and P25 (bending over) - in factor 1. This result supported the stability of a dimension assessing "motion sensitivity" represented by these 5 items. Disregarding F7 (difficulties in reading), these items encompass our second factor of the 4-factor solution (Table 2). The interest of clinicians in such a subscale is supported by Whitney et al. (2005) [28]. They investigated the usefulness of the above mentioned 5 items in predicting Benign Paroxysmal Positional Vertigo. Similar items also comprise the subscale "motion provoked dizziness" of a newly developed Vestibular Rehabilitation Benefit Questionnaire [29].

The interpretation of the dimensions of the DHI was mainly done by identifying the keywords of the questions and linking them with the ICF components respectively categories [4,23]. Furthermore we based our interpretation on the neurophysiology of the vestibular system, the described interactions of vestibular disorders and psychiatric co-morbidity [26,27] and the results of the previous factor analysis studies of the English [6], Spanish [7], and Dutch [8] version of the DHI (Additional file 2: Table S2). We are aware of the fact, however, that there might be different clinical interpretations of the retained factors.

Jacobson & Newman [1] distinguished between questions asking for a trigger of dizziness and unsteadiness (physical subscale) and questions asking for the consequences of these problems (Additional file 1: Table S1). According to the ICF model and our experience cause and effect can not strictly be separated. Therefore we do not think that our disregard of the syntax caused misinterpretations of the dimensions.

Future research should further investigate the construct validity of the newly defined dimensions of the DHI. It could be hypothesized that factor 3 moderately correlates with tests assessing walking ability e.g. the Dynamic Gait Index [30], the Functional Gait Assessment [31], or instrumented assessments of gait variability. It would also be interesting to find out how factor 2 correlates with objective measures of transfers or tests of functional capacity. Restructuring the DHI may allow a distinction among three patient groups: patients suffering from vertigo, dizziness and unsteadiness 1) mainly triggered by movements or effort, 2) by problems in the processing of afferent input, and 3) patients with emotional distress and restrictions in participation. This could refer to further specific assessments and support an early start of an effective treatment management.

## Conclusions

The Dizziness Handicap Questionnaire is a disease-specific health-related quality of life questionnaire. Like in previous studies the original subscale structure could not be supported, but the multidimensionality was obvious. The found 3-factor-solution showed comparable aspects with the results of previous factor analysis studies of the DHI. The retained factors could partly be interpreted with the ICF. The construct of the first factor could be supported by moderate associations with functional disability and non-somatic symptoms of anxiety and depression. In our opinion the 3 retained factors seem to be helpful for diagnostic or interventional decisions. Therefore a restructuring of the DHI might be discussed.

## Competing interests

The authors declare that they have no competing interests.

## Authors' contributions

AK contributed to the design of the survey. She conducted the statistical analysis and wrote the manuscript. CHGB contributed to the analysis of data and revised the article critically for its content. CJAWvG attributed to the design of the study, contributed to the interpretation of data and revised the article critically for its content. TG-J contributed to the design of the study, the acquisition of data, the interpretation of data and revised the article critically for its content. EDdB contributed to the analysis of data and revised the article critically for its content. DS co-initiated the study, contributed to the interpretation of data and revised the article critically for its content. All authors read and approved the final manuscript.

## Pre-publication history

The pre-publication history for this paper can be accessed here:

http://www.biomedcentral.com/1472-6815/10/3/prepub

## Supplementary Material

Additional file 1 **Linking the items of the DHI to ICF-categories**. This file represents the linking of each DHI item to ICF-labels and ICF-categories. Click here for file

Additional file 2 **Comparison of the 3-factor solution among different factor analysis studies of the Dizziness Handicap Inventory**. This file represents the results of the 3-factor solutions of the English, Spanish, Dutch, and German version of the DHI. Click here for file
