# Supplementary material for: Exploratory factor analysis of the Dizziness Handicap Inventory (German version)
Source: BMC Ear Nose Throat Disord. 2010 Mar 15;10:3. doi: 10.1186/1472-6815-10-3 (PMC2850321; doi:10.1186/1472-6815-10-3)
Supplement: Additional file 1 — Linking the items of the DHI to ICF-categories. This file represents the linking of each DHI item to ICF-labels and ICF-categories. [file 1472-6815-10-3-S1.DOC]

**Additional table 1** Linking the items of the DHI to ICF-categories

| 4 F | 3  F | Item | Questions | ICF-labels | ICF-categories |
| --- | --- | --- | --- | --- | --- |
| 1 | 1 | E 23 | Because of your problem, are you depressed? | emotional functions | b152 |
| 1 | 1 | E 2 | Because of your problem, do you feel frustrated? | emotional functions | b152 |
| 1 | 1 | F 24 | Does your problem interfere with your job or household responsibilities? | remunerative employment;  doing housework | d850  d640 |
| 1 | 1 | E 21 | Because of your problem, do you feel handicapped? | orientation to self;  experience of self and time functions | b11420  b180 |
| 1 | 1 | E 10 | Because of your problem, have you been embarrassed in front of others? | emotional functions;  societal attitudes | b152  e460 |
| 1 | 1 | F 6 | Does your problem significantly restrict your participation in social activities such as going out to dinner, going to movies, dancing, or to parties? | recreation and leisure | d920 |
| 1 | 1 | E 22 | Has your problem placed stress on your relationship with members of your family or friends? | family relationships;  informal social relationship | d760  d750 |
| 1 | 1 | F 3 | Because of your problem, do you restrict your travel for business or recreation? | using transportation;  driving | d470  d475 |
| 1 | 1 | E 18 | Because of your problem, is it difficult for you to concentrate? | focusing attention | d160 |
| 2 | 2 | P 13 | Does turning over in bed increase your problem? | changing basic body position | d410 |
| 2 | 2 | F 5 | Because of your problem, do you have difficulty getting into or out of bed? | lying down | d4100 |
| 2 | 2 | P 1 | Does looking up increase your problem? | watching | d110 |
| 2 | 2 | P 11 | Do quick movements of your head increase your problem? | control of voluntary movement functions;  structures of head and neck | b760  s710 |
| 2 | 2 | P 25 | Does bending over increase your problem? | bending | d4105 |
| 2 | 2 | F 7 | Because of your problem, do you have difficulty reading? | reading | d166 |
| 3 | 3 | P 17 | Does walking down a sidewalk increase your problem? | moving around outside the home and other buildings;  natural environment and human-made changes to environment | d4602  e298 |
| 3 | 3 | F 19 | Because of your problem, is it difficult for you to walk around your house in the dark? | moving around within the home;  light | d4600  e240 |
| 3 | 3 | E 15 | Because of your problem, are you afraid people may think you are intoxicated? | emotional functions;  vestibular functions of balance;  sensations associated with hearing and vestibular function;  societal attitudes | b152  b2351  b240  e460 |
| 3 | **2** | P 8 | Does performing more ambitious activities like sports, dancing, household chores increase your problem? | sports;  doing housework;  coordination of voluntary movement;  muscle power functions | d9201  d640  b7602  b730 |
| 3 | 3 | P 4 | Does walking down the aisle of a supermarket increase your problem? | moving around within buildings other than home;  design, construction and building products and technology of buildings for public use | d4601  e150 |
| 3 | 3 | F 12 | Because of your problem, do you avoid heights? | land forms;  design, construction and building products and technology of buildings for public use | e2100  e150 |
| 3 | **2** | F 14 | Because of your problem, is it difficult for you to do strenuous housework or yardwork? | doing housework;  muscle power functions;  muscle endurance functions | d640  b730  b740 |
| 4 | **3** | E 20 | Because of your problem, are you afraid to stay home alone? | emotional functions;  personal care providers and personal assistants;  friends | b152  e340  e320 |
| 4 | **3** | E 9 | Because of your problem, are you afraid to leave your home without having someone accompany you? | emotional functions;  personal care providers and personal assistants;  friends | b152  e340  e320 |
| 4 | **3** | F 16 | Because of your problem, is it difficult for you to go for a walk by yourself? | Moving around outside the home and other buildings;  personal assistants | d4602  e340 |

Abbreviations: 3F, 4F, indicates the components belonging to the 3- respectively 4-factor solution of the principal component analysis. Bold face indicates the differences between the 3- respectively 4-factor solution.

b- values of the International Classification of Functioning, Disability and Health (ICF) categorize body functions; d- values activities and participation; e- values environmental factors; s-values body structures. <http://apps.who.int/classifications/icfbrowser/>; 14.02.2010
