# Supplementary material for: Exploratory factor analysis of the Dizziness Handicap Inventory (German version)
Source: BMC Ear Nose Throat Disord. 2010 Mar 15;10:3. doi: 10.1186/1472-6815-10-3 (PMC2850321; doi:10.1186/1472-6815-10-3)
Supplement: Additional file 2 — Comparison of the 3-factor solution among different factor analysis studies of the Dizziness Handicap Inventory. This file represents the results of the 3-factor solutions of the English, Spanish, Dutch, and German version of the DHI. [file 1472-6815-10-3-S2.DOC]

**Additional table 2** Comparison of the 3-factor solution among different factor analysis studies of the Dizziness Handicap Inventory

| **DHI-G**  PCA/ oblique rotation  n = 194  49.2% of the variance are explained. | | | **DHI-D** (Vereeck et al.; 2007) [8]  PCA/ orthogonal rotation  n = 214  49.3% of the variance are explained. | | | **DHI-S** (Perez et al.; 2001) [7]  PCA/ orthogonal rotation  n = 337  48.3% of the variance are explained. | | | **DHI** (Asmundson et al.; 1999) [6]  PCA/ oblique rotation  N = 95  53.8% of the variance are explained**.** | | |
| --- | --- | --- | --- | --- | --- | --- | --- | --- | --- | --- | --- |
| **Factor 1: “Effect of dizziness and unsteadiness on emotion and participation.”** | | | **Factor 1: “Functional disability – inability to be an active member of the society.”*** | | | **Factor 1: “Vestibular handicap -**  **Social or cultural consequences of dizziness and unsteadiness.”** | | | **Factor 1: “Disability in activities of daily living.”** | | |
| E 23 | **0.792** | feeling depressed | E 23 | **0.696** | feeling depressed | E 23 | 0.590 | feeling depressed | E 23 | 0.48 | feeling depressed |
| E 2 | **0.757** | feeling frustrated | E 2 | 0.608 | feeling frustrated | E 2 | 0.635 | feeling frustrated | E 2 | 0.52 | feeling frustrated |
| E 21 | **0.653** | feeling handicapped | E 21 | **0.780** | feeling handicapped | E 21 | **0.742** | feeling handicapped | E 21 | 0.65 | feeling handicapped |
| F 24 | 0.652 | job/ house responsibilities | F 24 | 0.447 | job/ house responsibilities | F 24 | **0.688** | job/ house responsibilities | F 24 | 0.72 | job/ house responsibilities |
| E 10 | 0.622 | embarrassed in front of others | E 10 | **0.703** | embarrassed in front of others | E 10 | 0.559 | embarrassed in front of others |  |  |  |
| F 6 | 0.567 | restriction of social activities | F 6 | 0.670 | restriction of social activities | F 6 | 0.691 | restriction of social activities | F 6 | 0.55 | restriction of social activities |
| E 22 | 0.563 | stressed relationships |  |  |  | E 22 | 0.479 | stressed relationships | E 22 | 0.52 | stressed relationships |
| F 3 | 0.463 | restriction of travel | F 3 | 0.640 | restriction of travel | F 3 | 0.663 | restriction of travel |  |  |  |
| E 18 | 0.397 | difficulties in concentrating |  |  |  | E 18 | 0.616 | difficulties in concentrating | E 18 | **0.84** | difficulties in concentrating |
|  |  |  |  |  |  |  |  |  |  |  |  |
|  |  |  |  |  |  | F 14 | **0.733** | strenuous housework | F 14 | 0.59 | strenuous housework |
|  |  |  |  |  |  |  |  |  | P 8 | 0.61 | ambitious activities like sports |
|  |  |  | E 9 | 0.679 | afraid of leaving home alone | E 9 | 0.626 | afraid of leaving home alone |  |  |  |
|  |  |  | F 16 | 0.617 | walking by yourself | F 16 | 0.621 | walking by yourself |  |  |  |
|  |  |  | E 20 | 0.543 | afraid to stay home alone | E 20 | 0.536 | afraid to stay home alone |  |  |  |
|  |  |  | P 17 | 0.425 | walking down a sidewalk |  |  |  |  |  |  |
|  |  |  |  |  |  |  |  |  | F 19 | 0.42 | walking around in dark |
|  |  |  | E 15 | 0.495 | afraid of appearing intoxicated |  |  |  |  |  |  |
|  |  |  |  |  |  |  |  |  | P 25 | **0.74** | bending over |
|  |  |  |  |  |  |  |  |  | P 11 | **0.75** | quick head movements |
|  |  |  |  |  |  |  |  |  | P 1 | 0.61 | looking up |
|  |  |  |  |  |  | F 7 | 0.448 | difficulties in reading | F 7 | 0.65 | difficulties in reading |

| **Factor 2: Specific activities/**  **movements or effort provoking dizziness or unsteadiness.”** | | | **Factor 2: “Vestibular disability – motion sensitivity.”*** | | | **Factor 2: “Vestibular disability –**  **Limitation in performance of daily activities.”** | | | **Factor 2: “Postural difficulties.”** | | |
| --- | --- | --- | --- | --- | --- | --- | --- | --- | --- | --- | --- |
| P 13 | **0.747** | turning over in bed | P 13 | 0.532 | turning over in bed | P 13 | **0.768** | turning over in bed | P 13 | **0.75** | turning over in bed |
| P 11 | **0.726** | quick head movements | P 11 | **0.700** | quick head movements | P 11 | 0.602 | quick head movements |  |  |  |
| P 1 | **0.707** | looking up | P 1 | **0.679** | looking up |  |  |  |  |  |  |
| F 5 | 0.683 | getting into or out of bed | F 5 | 0.510 | getting into or out of bed | F 5 | **0.692** | getting into or out of bed | F 5 | **0.81** | getting into or out of bed |
| P 25 | 0.618 | bending over | P 25 | **0.669** | bending over | P 25 | **0.691** | bending over |  |  |  |
| P 8 | 0.439 | ambitious activities like sports | P 8 | 0.660 | ambitious activities like sports | P 8 | 0.439 | ambitious activities like sports |  |  |  |
| F 14 | 0.410 | strenuous housework | F 14 | 0.522 | strenuous housework |  |  |  |  |  |  |
| F 7 | 0.253 | difficulties in reading |  |  |  |  |  |  |  |  |  |
|  |  |  | F 12 | 0.551 | avoid heights |  |  |  |  |  |  |

| **Factor 3: “Walking ability and feeling of postural stability in relation to contextual factors.”** | | | | **Factor 3: “Vestibular disability – visuo-vestibular disability”*** | | | **Factor 3: “Visuo-vestibular disability”** | | | | | **Factor 3: “Phobic avoidance.”** | | |
| --- | --- | --- | --- | --- | --- | --- | --- | --- | --- | --- | --- | --- | --- | --- |
| P 17 | **0.713** | walking down a sidewalk | |  |  |  | P 17 | **0.618** | | walking down a sidewalk | | P 17 | **0.74** | walking down a sidewalk |
| F 19 | **0.701** | walking around in dark | | F 19 | 0.438 | walking around in the dark | F 19 | 0.374 | | walking around in dark | |  |  |  |
| F 16 | **0.623** | walking by yourself | |  |  |  |  |  | |  | | F 16 | **0.82** | walking by yourself |
| E 15 | 0.580 | afraid of appearing intoxicated | |  |  |  | E 15 | **0.597** | | afraid of appearing intoxicated | | E 15 | 0.56 | afraid of appearing intoxicated |
| E 9 | 0.572 | afraid of leaving home alone | |  |  |  |  |  | |  | | E 9 | **0.69** | afraid of leaving home alone |
| P 4 | 0.553 | walking down a supermarket aisle | P 4 | | 0.493 | walking down a supermarket aisle | P 4 | | 0.538 | | walking down a supermarket aisle | P 4 | 0.60 | walking down a supermarket aisle |
| F 12 | 0.543 | avoid heights |  | |  |  | F 12 | | **0.608** | | avoid heights | F 12 | 0.50 | avoid heights |
| E 20 | 0.47 | afraid to stay home alone |  | |  |  |  | |  | |  | E 20 | 0.47 | afraid to stay home alone |
|  |  |  | E 18 | | **0.734** | difficulties in concentrating |  | |  | |  |  |  |  |
|  |  |  | E 22 | | **0.522** | stressed relationships |  | |  | |  |  |  |  |
|  |  |  |  | |  |  |  | |  | |  | F 3 | 0.47 | restriction of travel |
|  |  |  |  | |  |  |  | |  | |  | E 10 | 0.43 | embarrassed in front of others |
|  |  |  | F 7 | | **0.655** | difficulties in reading |  | |  | |  |  |  |  |
|  |  |  |  | |  |  | P 1 | | 0.572 | | looking up |  |  |  |

Abbreviations: DHI-G indicates the German version, DHI-D, the Dutch version, DHI-S, the Spanish version, DHI, the original version of the DHI. E indicates emotional subscale of the DHI, F, functional subscale; P, physical subscale; PCA, principal component analysis.

* The titles of the factors retained by Vereeck et al. (2007) belong originally to their 4-factor solution. To facilitate the comparison, the order of the items of the previous factor analytic studies was adapted to the order of items in our 3-factor solution. Bold face indicates the three highest factor loadings per factor.
